# Juvenile Social Isolation Enhances the Activity of Inhibitory Neuronal Circuits in the Medial Prefrontal Cortex

**DOI:** 10.3389/fncel.2020.00105

**Published:** 2020-05-12

**Authors:** Kazuhiko Yamamuro, Hiroki Yoshino, Yoichi Ogawa, Kazuya Okamura, Yosuke Nishihata, Manabu Makinodan, Yasuhiko Saito, Toshifumi Kishimoto

**Affiliations:** ^1^Department of Psychiatry, Nara Medical University, Kashihara, Japan; ^2^Department of Neurophysiology, Nara Medical University, Kashihara, Japan

**Keywords:** prefrontal cortex, inhibitory circuit, neurodevelopment, social isolation, pyramidal cell, fast-spiking cell

## Abstract

During brain development, the design of primary neural networks is primarily determined by environmental stimuli after their formation. In particular, the juvenile period is critical, during which neuronal circuits that consist of both excitatory and inhibitory neurons are remodeled by experience. Social isolation during the juvenile period profoundly affects brain development and contributes to the development of psychiatric disorders. We previously reported that 2 weeks of social isolation after weaning reduced excitatory synaptic inputs and intrinsic excitability in a subtype of layer 5 pyramidal cells, which we defined as prominent h-current (PH) cells, in the medial prefrontal cortex (mPFC) in mice. However, it remains unclear how juvenile social isolation affects inhibitory neuronal circuits that consist of pyramidal cells and interneurons. We found that 2 weeks of social isolation after weaning increased inhibitory synaptic inputs exclusively onto PH cells with a concomitant deterioration of action potential properties. Although social isolation did not alter the inhibitory synaptic release mechanisms or the number of inhibitory functional synapses on PH cells, we found that it increased the intrinsic excitability of fast-spiking (FS) interneurons with less excitatory synaptic inputs and more h-current. Our findings indicate that juvenile social isolation enhances the activity of inhibitory neuronal circuits in the mPFC.

## Introduction

There are certain distinct critical periods during programmed development. Early-life experiences can activate intrinsic mechanisms during these critical periods to increase neuronal plasticity. For example, complete darkness after birth has been reported to limit visual experiences, which leads to an immature visual cortex and a prolonged onset of the visual cortex’s critical period (Daw et al., 1995). Therefore, experience is crucial for brain function refinement and young animals, including humans, adapt to the environments in which they live. Neuronal plasticity becomes limited after brain maturation. Therefore, lack of early-life remodeling and consolidation of circuits might cause dysregulated information processing, which is associated with neurodevelopmental psychiatric disorders, such as schizophrenia and autism spectrum disorder (LeBlanc and Fagiolini, 2011; Takesian and Hensch, 2013; Nelson and Valakh, 2015).

Juvenile social experiences substantially affect brain function and behavior in humans and other species (Freedman et al., 1961; Chugani et al., 2001; Bick et al., 2017). Several studies have reported that aberrant juvenile social experiences have enduring effects on brain structure and function that persist into adulthood (Weaver et al., 2004; Eluvathingal et al., 2006; Makinodan et al., 2012; Yamamuro et al., 2018). It is important to note that these sequelae are not reversed by subsequent foster care in humans or subsequent environmental enrichment in rodents (Chugani et al., 2001; Eluvathingal et al., 2006; Makinodan et al., 2012; Yamamuro et al., 2018). These findings suggest that the juvenile period is critical for brain development promoted by social experience.

We previously studied the effect of juvenile social isolation on the synaptic and intrinsic excitability of layer (L) 5 pyramidal cells in the medial prefrontal cortex (mPFC) in mice. L5 pyramidal cells in the PFC can be classified into at least two subtype populations. One subtype projects to subcortical regions, has thicker apical dendrites, more primary branches, and displays prominent hyperpolarization-activated cation currents (Ih). Conversely, the other subtype projects to the contralateral cortex, has thinner apical dendrites, and lacks prominent Ih (Dembrow et al., 2010; Gee et al., 2012; Lee et al., 2014). We defined L5 pyramidal cells with prominent Ih as PH cells and those without as non-PH cells. We found that 2 weeks of social isolation after weaning reduced excitatory synaptic inputs and the excitability of PH cells (Yamamuro et al., 2018).

For optimized neuronal circuit function in behavior, a proper balance between excitation and inhibition in the circuit is critical (Yizhar et al., 2011). Neurodevelopmental disorders, such as schizophrenia, have underlying imbalances of excitation and inhibition in the neuronal circuits (Gonzalez-Burgos et al., 2015; Foss-Feig et al., 2017). However, it remains unclear how juvenile social isolation affects inhibitory neuronal circuits, including inhibitory synaptic inputs to L5 pyramidal cells and interneurons as upstream inhibitory synaptic transmission.

It has been reported that γ-aminobutyric acid (GABA)-mediated inhibition influences cortical network activity and maturation (Buzsáki et al., 2013). In the rodent neocortex, inhibitory synaptogenesis primarily occurs postnatally and rapidly reaches adult-like inhibitory synapse density levels prior to adolescence (Micheva and Beaulieu, 1996; De Felipe et al., 1997). Moreover, the time course for functional maturation of GABAergic synapses in rodents is similar to that of inhibitory synaptogenesis; specifically, the inhibitory postsynaptic current (IPSC) frequency only becomes significant after birth with the IPSCs exhibiting adult-like properties prior to adolescence onset (Le Magueresse and Monyer, 2013). Therefore, the juvenile period might be a sensitive period during which social experience affects inhibitory synaptic transmission.

The neocortex contains a diverse GABAergic interneuron population that collectively regulates the activity of principal neurons. Within this population, a subgroup of interneurons with a fast-spiking (FS) phenotype and powerful perisomatic-targeting inhibitory synapses on principal neurons is thought to contribute to the generation of oscillatory network activity, which is important for cognitive function (Freund and Katona, 2007; Lewis et al., 2011). FS interneurons frequently express parvalbumin (PV), which is a calcium-binding protein, and display several distinct electrophysiological properties, including low input resistance, short membrane time constants, narrow action potentials, and a high maximum firing frequency in response to depolarizing current pulses (González-Burgos et al., 2005; Doischer et al., 2008). Interestingly, FS PV interneurons preferentially inhibit L5 pyramidal cells with a prominent Ih compared with L5 pyramidal cells without a prominent Ih (Lee et al., 2014). Subgroups of GABAergic interneurons in the hippocampus and neocortex have been reported to express cholecystokinin, somatostatin (SST), and vasointestinal peptide (Freund and Buzsáki, 1996; Markram et al., 2004; Tricoire et al., 2011). These interneurons typically produce broader action potentials at a lower frequency than those produced by FS interneurons. Although there are functional differences between the subtypes of these interneurons, they are collectively classified as non-FS interneurons based on their morphological and electrophysiological parameters (Kawaguchi and Kondo, 2002; Ascoli et al., 2008; Andersson et al., 2012).

To investigate the effect of juvenile social isolation on inhibitory synaptic inputs onto L5 pyramidal cells and excitability of L5 interneurons, which might output inhibitory synaptic transmission to L5 pyramidal cell, we used the whole-cell patch clamp technique to record IPSCs on L5 pyramidal cells (PH and non-PH cells) and the excitabilities of interneurons (FS cells and non-FS cells), and compared them between group-housed (GH) and isolate-housed mice.

## Materials and Methods

### Mice and Housing Conditions

All the study experiments were approved by the animal care and use committee of Nara Medical University and were conducted according to its guidelines. We used male C57/BL6 mice for all experiments, which were maintained on a fixed 12-h light-dark cycle. After weaning on postnatal day (P) 21, we randomly divided every four male littermates as follows: one was isolated and three were group-reared. The isolated mouse was individually housed from P21 to P35 (early isolation; E-IH) or P35 to P49 (late isolation; L-IH). We considered the GH mice as the typically developing mice. During the non-isolated period, we housed each isolated mouse with its three littermates from P63 to P70.

### Electrophysiology

We prepared brain slices, including the medial frontal cortex (prelimbic cortex), from 63- to 70-day-old mice. We deeply anesthetized the mice using isoflurane and decapitated them. We quickly removed the brain and immersed it in an ice-cold sucrose-based solution bubbled with a mixture of 95% O_2_/5% CO_2_ gas. The solution contained the following (in mM): 230 sucrose, 2.5 KCl, 25 NaHCO_3_, 1.25 NaH_2_PO_4_, 0.5 CaCl_2_, 10 MgSO_4_, and 10 D-glucose. We sectioned the frontal cortex into 330 μm-thick slices in the coronal plane using a vibrating tissue slicer (Vibratome 1000 Plus 102, Pelco International, Redding, CA, USA). We incubated the slices for at least 60 min in a chamber filled with standard artificial cerebrospinal fluid (ACSF; mM) and continuously bubbled it with a gas mixture at 32°C containing (in mM) the following: 125 NaCl, 2.5 KCl, 25 NaHCO_3_, 1.25 NaH_2_PO_4_, 2.0 CaCl_2_, 1.0 MgCl_2_, and 25 D-glucose. Next, we maintained the slices in ACSF at 25°C. Following incubation, we transferred the submerged slices to a recording chamber and superfused them at a flow rate of 2 ml per min with the ACSF saturated with the aforementioned gas mixture at 32°C.

We visualized the cells using an upright microscope (BW50WI, Olympus, Japan) equipped with infrared illumination and differential interference contrast video microscopy. We voltage- or current-clamped the L5 neurons of prelimbic cortex in the conventional whole-cell configuration using a Multiclamp 700A amplifier (Axon Instruments). We pulled patch pipettes from borosilicate glass and filled them with an intracellular solution containing the following (in mM): 141 K-gluconate, 4 KCl, 2 MgCl_2_, 2 Mg-ATP, 0.3 Na-GTP, 0.2 EGTA, and 10 HEPES with a pH of 7.25 achieved using KOH to record excitatory postsynaptic current (EPSC) from interneurons. To record the IPSC from pyramidal cells, we used an intracellular solution containing the following (in mM) 95 K-gluconate, 50 KCl, 2 MgCl_2_, 2 Mg-ATP, 0.3 Na-GTP, 0.2 EGTA, and 10 HEPES with pH 7.25 achieved using KOH. All the recorded membrane potentials were corrected for 13 mV liquid junction potential that was measured as previously described (Neher, 1992). We controlled data acquisition and stimulation using Signal 4 software with Power 1401 interface equipment (Cambridge Electronic Design).

### Neuron Classification

We visually identified the pyramidal cells and interneurons prior to the aforementioned recordings. To distinguish FS interneurons from non-FS interneurons, depolarizing current steps were used to analyze the firing response of each interneuron. We determined the single spike properties for spikes elicited by near-threshold current injections, and quantified spike-frequency adaptation as the ratio between the last and first inter-spike interval in spike trains evoked by 500-ms depolarizing steps. Cells were classified FS if they exhibited the following characteristics: (1) narrow spikes (duration at half peak amplitude ≤ 0.6 ms); (2) large after hyperpolarizing potentials (amplitude ≥ 15 mV); and (3) no significant spike-frequency adaptation (adaptation ratio ≤ 1.2). These criteria might have led to the exclusion of some FS neurons but ensured that cells that were included were FS cells (Supplementary Figures S1A,B). As previously reported (Kawaguchi and Kubota, 1997; Galarreta and Hestrin, 2002; Pawelzik et al., 2002), there is a high correlation between FS electrical properties and PV expression.

### Voltage Clamp Recordings

For voltage-clamp recordings, pipette capacitance was compensated while series resistance was continuously monitored and not compensated. We only used recordings with a stable series resistance of <20 MΩ in the subsequent analyses. Current signals were low-pass filtered at 600 Hz and digitized at a sampling frequency of 10 kHz. We held the pyramidal cells at −70 mV to record the postsynaptic current. As previously described, we used high-chloride intracellular solution to record IPSC and normal chloride intracellular solution to record EPSC. We recorded spontaneous inhibitory postsynaptic currents (sIPSCs) in ACSF with 10 μM CNQX, an AMPA/kainite receptor antagonist, and recorded spontaneous excitatory postsynaptic currents (sEPSCs) in ACSF lacking GABAergic antagonists to maintain both excitatory and inhibitory neuronal activity in the slice. Moreover, we recorded tetrodotoxin (TTX)-resistant miniature IPSCs (mIPSCs) in the presence of 10 μM CNQX and 1 μM TTX. We also recorded miniature EPSCs (mEPSCs) in the presence of 10 μM gabazine and 1 μM TTX. To record evoked IPSCs (eIPSCs), cells were electrically stimulated using a pipette fabricated with theta-type capillary glass pulled to an open tip diameter of 3–5 μm and filled with ACSF. The eIPSC was recorded in ACSF containing 10 μM CNQX. We connected a silver bipolar electrode inserted into the theta pipette to an A365 stimulus isolation unit (World Precision Instruments) that was commanded using transistor-transistor logic compatible pulses. The stimulation pipette was placed in L5 at 100–200 μm from the soma of the recorded pyramidal cell and in L3 apically from the soma of the recorded pyramidal cell and a constant-current stimulus (50 μs in duration) at 0.2 Hz was delivered.

### Current-Clamp Recordings

For current-clamp recordings, we monitored and canceled series resistance using a bridge circuit; further, pipette capacitance was compensated. Voltage signals were low-pass filtered at 10 kHz and digitized at 20 kHz. The baseline membrane potential was maintained at approximately –70 mV through current injection. To examine action potential and subthreshold membrane properties, we recorded the membrane potential responses to hyperpolarizing and depolarizing current pulses (500 ms in duration).

We assessed the Ih magnitude by measuring the voltage sag at hyperpolarization induced by a –50 pA current injection and calculated the sag ratio (sag-R) as previously described (Yamamuro et al., 2018); and defined cells with >5% sag-R as prominent Ih (PH) cells and the other cells as non-PH cells (Yamamuro et al., 2018). There have been recent reports on the definitions of L5 pyramidal cell subtypes using a similar method with the combined current values, including Ih-induced sag (Lee et al., 2014; Yamamuro et al., 2018).

### Data Analyses

We analyzed sIPSCs and mIPSCs using Mini Analysis software (Synaptosoft). For each cell, we detected and analyzed all events for 5 min (sPSC) or 10 min (mPSC), and used scatter plots of the intrinsic membrane property data to allow the explicit display of the variations.

We presented the data as mean ± standard error of the mean through standard bar charts or line plots. Statistical analyses were performed using Prism (Graphpad). We determined statistical differences using student’s *t*-tests, Mann–Whitney test, or two-factor analyses of variance (ANOVAs) followed by Tukey’s honest significant difference test. For repeated-measures data, multivariate ANOVAs was used when the variance/covariance matrix was not circular or when the assumption of equality between the variance/covariance matrices was rejected. Between-group differences in the means were considered significant at *p* < 0.05.

## Results

### Juvenile Social Isolation Increased Inhibitory Synaptic Inputs Onto PH Cells

**Figure 1:**
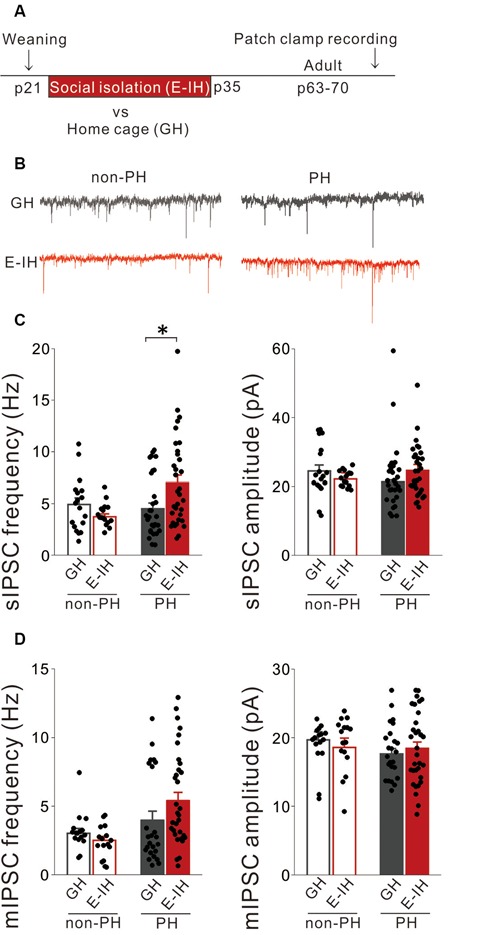
Juvenile social isolation increased inhibitory synaptic inputs only onto prominent h-current (PH) cells but not onto non-PH cells. **(A)** Timeline showing weaning on postnatal day (P) 21 and the two subsequent weeks of early isolate-housing (E-IH) followed by re-housing or group housing (GH). We conducted whole-cell patch clamp recordings of pyramidal cells in the medial prefrontal cortex (mPFC) of the mice from P63 to P70. **(B)** Representative traces showing spontaneous inhibitory postsynaptic currents (sIPSCs) recorded from PH (right) and non-PH cells (left) in the GH (upper) and E-IH (lower) mice. **(C)** Left: for PH cells, the E-IH mice had a significantly higher sIPSC frequency than that in the GH mice (Tukey’s HSD test: *p < 0.05). However, for non-PH cells, there were no significant between-group differences in the sIPSC frequency (2-way analysis of variance (ANOVA), effect of housing, *F*_(1,93)_ = 0.866, *p* = 0.355, effect of h-current, *F*_(1,93)_ = 4.239, *p* = 0.042, housing × h-current, *F*_(1,93)_ = 7.203, *p* = 0.008). Right: for both PH and non-PH cells, there was no between-group difference in the sIPSC amplitude (2-way ANOVA, effect of housing, *F*_(1,93)_ = 0.067, *p* = 0.797, effect of h-current, *F*_(1,93)_ = 0.124, *p* = 0.725, housing × h-current, *F*_(1,93)_ = 2.459, *p* = 0.120; number of cells: 18 non-PH and 29 PH from six GH mice: 16 non-PH and 34 PH from six E-IH mice). **(D)** Left: there was no significant between-group difference in the miniature IPSC (mIPSC) frequency in either PH or non-PH cells (2-way ANOVA, effect of housing, *F*_(1,84)_ = 0.531, *p* = 0.468, effect of h-current, *F*_(1,84)_ = 9.366, **p = 0.003, housing × h-current, *F*_(1,84)_ = 2.409, *p* = 0.125). Right: there was no significant between-group difference in the mIPSC amplitude in PH or non-PH cells (2-way ANOVA, effect of housing, *F*_(1,84)_ = 0.024, *p* = 0.878, effect of h-current, *F*_(1,84)_ = 1.274, *p* = 0.262, housing × h-current, *F*_(1,84)_ = 0.987, *p* = 0.323; number of cells: 16 non-PH and 16 PH from six GH mice: 24 non-PH and 32 PH from six E-IH mice).

We previously reported that juvenile social isolation decreased excitatory synaptic inputs onto PH cells (Yamamuro et al., 2018). Synaptic connections and intrinsic neuronal excitability are controlled by homeostatic mechanisms that maintain stable neuronal function (Davis, 2013). Therefore, decreases in excitatory synaptic inputs onto PH cells induced by social isolation might be compensated for by decreased inhibitory synaptic inputs onto PH cells. Alternatively, since visual deprivation during the critical period enhances GABAergic inhibition in the visual cortex in mice (Nahmani and Turrigiano, 2014; Kannan et al., 2016), the lack of social experience might potentiate GABAergic inhibition in the mPFC. To investigate these possibilities, we recorded the sIPSCs of L5 pyramidal cells in the mPFC during adulthood and determined the cell-type specific effects of juvenile social isolation from P21 to P35 (E-IH) on inhibitory synaptic inputs onto PH cells and non-PH cells (Figure 1A). Isolated mice exhibited a significant increase in the frequency of sIPSCs on PH cells but not on non-PH cells (Figures 1B,C; left). There were no significant differences in the sIPSC amplitudes in either PH or non-PH cells between GH mice and E-IH mice (Figures 1B,C; right). Moreover, we analyzed the mIPSCs in L5 pyramidal cells and observed that social isolation had no effect on mIPSC frequency or amplitude in either PH or non-PH cells (Figure 1D). These findings indicated that social isolation enhanced inhibitory synaptic inputs onto PH cells, which could subsequently lower the excitability of PH cells. We observed that PH cells in the E-IH mice fired at a significantly lower spike frequency during a 100-pA current injection (Supplementary Figures S2A,C); further, they had a higher spike threshold (Supplementary Figures S2A,D) than that observed in the GH mice, which is consistent with previous findings (Yamamuro et al., 2018). Although social isolation did not affect the spike amplitudes (Supplementary Figures S2A,B), sag-R (Supplementary Figure S2E), or input resistance (Supplementary Figure S2F), we found a negative correlation between the sag-R and input resistance in both the GH and E-IH groups (Supplementary Figure S2G). These results suggest that juvenile social isolation increased inhibitory inputs and lowered the excitability of PH cells but not non-PH cells.

### The Early Juvenile Period Is Critical for Social Isolation to Affect Inhibitory Synaptic Inputs Onto and the Intrinsic Excitability of PH Cells

**Figure 2:**
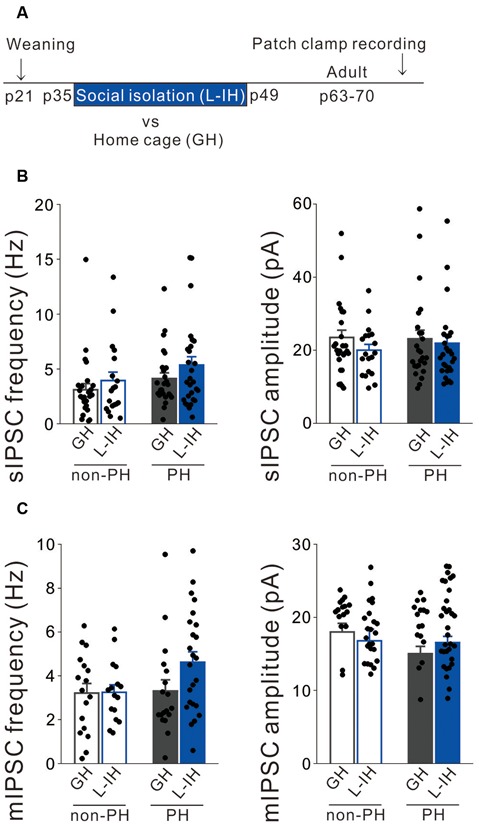
Social isolation did not affect inhibitory synaptic inputs onto L5 pyramidal cells after the critical period. **(A)** Timeline showing weaning on postnatal day (P) 21 and the 2 weeks of late isolate-housing (L-IH) from P35 followed by re-housing or group housing (GH). We conducted whole-cell patch clamp recordings from mPFC pyramidal cells in adulthood. **(B)** Left: there were no significant between-group differences in the spontaneous inhibitory postsynaptic current (sIPSC) frequency in either prominent h-current (PH) or non-PH cells (2-way ANOVA, effect of housing, *F*_(1,94)_ = 2.494, *p* = 0.118, effect of h-current, *F*_(1,94)_ = 3.424, *p* = 0.067, housing × h-current, *F*_(1,94)_ = 0.099, *p* = 0.754). Right: there was no between-group difference in sIPSC amplitude in either PH or non-PH cells (2-way ANOVA, effect of housing, *F*_(1,94)_ = 1.3, *p* = 0.257, effect of h-current, *F*_(1,94)_ = 0.135, *p* = 0.714, housing × h-current, *F*_(1,94)_ = 0.304, *p* = 0.583; number of cells: 26 non-PH and 25 PH from six GH mice: 19 non-PH and 28 PH from six L-IH mice). **(C)** Left: there were no significant between-group differences in the miniature IPSC (mIPSC) frequency in either PH or non-PH cells (2-way ANOVA, effect of housing, *F*_(1,75)_ = 2.035, *p* = 0.158, effect of h-current, *F*_(1,75)_ = 2.437, *p* = 0.123, housing × h-current, *F*_(1,75)_ = 1.851, *p* = 0.178). Right: there was no between-group difference in the mIPSC amplitude in either PH or non-PH cells (2-way ANOVA, effect of housing, *F*_(1,75)_ = 0.108, *p* = 0.744, effect of h-current, *F*_(1,75)_ = 3.429, *p* = 0.068, housing × h-current, *F*_(1,75)_ = 2.725, *p* = 0.103; number of cells: 17 non-PH and 22 PH from six GH mice: 16 non-PH and 24 PH from six L-IH mice).

We previously reported that late social isolation (P35 to P49) did not affect the excitatory synaptic inputs onto and excitability of PH cells (Yamamuro et al., 2018). Similarly, we hypothesized that late social isolation did not affect inhibitory synaptic inputs onto PH cells. Therefore, we investigated whether late social isolation (L-IH; P35 to P49) affected the inhibitory synaptic inputs and action potential properties in L5 pyramidal cells of the mPFC using the previously used protocol (Yamamuro et al., 2018; Figure 2A). Similarly to our previous report (Yamamuro et al., 2018), we observed no differences in the spike amplitude (Supplementary Figure S3A), spike frequency during a depolarizing current injection (Supplementary Figure S3B), or spike threshold (Supplementary Figure S3C) between the L-IH and GH mice in either PH or non-PH cells. Although social isolation did not affect the sag-R or input resistance (Supplementary Figures S3D,E), we observed a negative correlation between the sag-R and input resistance in both the GH and E-IH mice (Supplementary Figure S3F). Further, we did not observe any significant differences in the frequency or amplitude of sIPSCs in PH cells and non-PH cells between the L-IH and GH mice (Figure 2B) or in the frequency or amplitude of mIPSCs (Figure 2C). These results indicate that P21 to P35 is a sensitive developmental period during which social isolation specifically affects inhibitory synaptic inputs and excitability of pyramidal cells in the mPFC.

### Social Isolation Did Not Affect the Number of Functional Inhibitory Synapses or Inhibitory Neurotransmitter Release Mechanisms in Perisomatic and Apical Dendritic Inhibition of PH Cells

**Figure 3:**
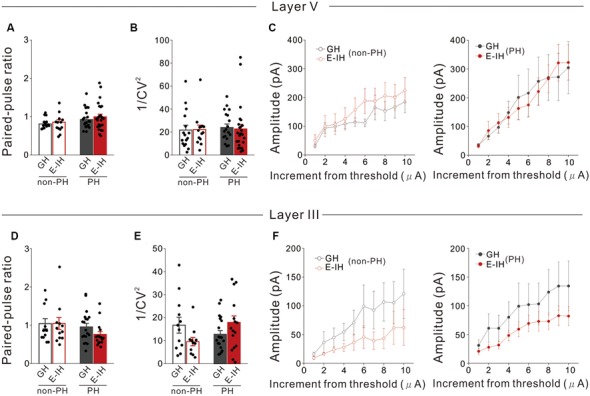
Social isolation did not affect the number of functional inhibitory synapses onto or presynaptic inhibitory neurotransmitter release mechanisms in L5 pyramidal cells in the perisomatic and apical dendritic site. **(A)** There were no significant between-group differences in the paired-pulse ratio (PPR) in prominent h-current (PH) and non-PH cells (2-way ANOVA, effect of housing, *F*_(1,71)_ = 0.729, *p* = 0.396, effect of h-current, *F*_(1,71)_ = 5.458, *p* = 0.022, housing × h-current, *F*_(1,71)_ = 0.107, *p* = 0.745; number of cells: 17 non-PH and 20 PH from five GH mice: 13 non-PH and 25 PH from five E-IH mice). **(B)** There were no significant between-group differences in 1/coefficient of variation (CV)^2^ in PH or non-PH cells (2-way ANOVA, effect of housing, *F*_(1,69)_ = 0.0087, *p* = 0.926, effect of h-current, *F*_(1,69)_ = 0.186, *p* = 0.668, housing × h-current, *F*_(1,69)_ = 0.148, *p* = 0.702; number of cells: 16 non-PH and 18 PH from five GH mice: 14 non-PH and 25 PH from five E-IH mice). **(C)** Line plots showing the relationship between the stimulus intensity (increment from the threshold current) and evoked excitatory postsynaptic current (eEPSC) amplitude in non-PH (left) and PH (right) cells. There were no significant between-group differences in eEPSC amplitudes across the stimulation intensities in non-PH (MANOVA, housing × current step interaction, *F*_(9,250)_ = 0.176, *p* = 0.996; left) and PH cells (MANOVA, housing × current step interaction, *F*_(9,340)_ = 0.178, *p* = 0.996; right; number of cells: 17 non-PH and 14 PH from five GH mice : 10 non-PH and 22 PH from five E-IH mice). **(D)** There were no significant between-group differences in the PPR in PH or non-PH cells (2-way ANOVA, effect of housing, *F*_(1,56)_ = 0.724, *p* = 0.399, effect of h-current, *F*_(1,56)_ = 2.804, *p* = 0.100, housing × h-current, *F*_(1,56)_ = 0.823, *p* = 0.363; number of cells: 12 non-PH and 19 PH from five GH mice: 12 non-PH and 17 PH from five E-IH mice). **(E)** There was a significantly higher 1/ CV^2^ in PH cells than that in non-PH cells among the early isolation (E-IH) mice (2-way ANOVA, effect of housing, *F*_(1,54)_ = 0.160, *p* = 0.691, effect of h-current, *F*_(1,54)_ = 0.725, *p* = 0.398, housing × h-current, *F*_(1,54)_ = 5.811, *p* = 0.019) but they did not differ in the group housing (GH) mice (number of cells: 17 non-PH and 14 PH from five GH mice: 10 non-PH and 22 PH from five E-IH mice). **(F)** Line plots showing the relationship between stimulus intensity and eEPSC amplitude in non-PH (left) and PH (right) cells. There were no significant between-group differences in the eEPSC amplitude across stimulation intensities in both non-PH (MANOVA, housing × current step interaction, *F*_(9,190)_ = 0.172, *p* = 0.997; left) and PH cells (MANOVA, housing × current step interaction, *F*_(9,340)_ = 0.0055, *p* = 0.999; right; number of cells: 11 non-PH and 19 PH from five GH mice: 10 non-PH and 17 PH from five E-IH mice).

We previously reported that social isolation during the critical juvenile period reduced the sEPSC and mEPSC frequency potentially by reducing functional excitatory synapses on PH cells only (Yamamuro et al., 2018). However, in the present study, we found that social isolation increased the sIPSC frequency but not the mIPSC frequency. Since social isolation did not affect the mIPSC frequency, it is not plausible that the mechanisms through which social isolation increases the sIPSC frequency in PH cells involve increased functional inhibitory synapses or inhibitory neurotransmitter release onto PH cells. Therefore, to verify this assumption, we measured eIPSCs evoked by stimulation electrodes placed in L5 lateral to the soma of the recorded pyramidal cell, which stimulates perisomatic inhibition, and determined the paired-pulse ratio (PPR) and coefficient of variation (CV) of the amplitude. We calculated the inverse squared value of CV (1/CV^2^) and used it as a standard measure for evaluating inhibitory synaptic function (Kullmann, 1994). PPR alterations indicate changes in neurotransmitter release mechanisms at presynaptic terminals (Zucker and Regehr, 2002) while 1/CV^2^ alterations suggest changes in either the probability of transmitter release, number of functional synapses, or both (Kullmann, 1994). We found no significant differences in the PPR in either PH or non-PH cells between the GH and E-IH mice (Figure 3A), which indicated that juvenile social isolation did not affect the mechanisms of presynaptic transmitter release. Moreover, there were no significant differences in the 1/CV^2^ in either PH or non-PH cells between the GH and E-IH mice (Figure 3B). To further investigate whether social isolation affected the number of functional inhibitory synapses, we analyzed the intensity-amplitude curves recorded from IPSCs evoked by electrical stimulation using current that was incrementally increased at 0.5-μA steps from the threshold intensity. We observed no significant differences in the eIPSC in either PH or non-PH cells between the GH and E-IH mice (Figure 3C). These results indicate that juvenile social isolation does not increase the number of functional inhibitory synapses or alter the presynaptic transmitter release mechanisms in perisomatic inhibition of PH cells. However, it is also possible that increased sIPSC frequency resulting from social isolation could involve an increased number of inhibitory synapses or altered neurotransmitter release mechanisms in inhibitory synaptic inputs in the distal site of the apical dendrites in PH cells. Therefore, we placed a stimulation electrode in L3, which includes the apical dendrites, of an L5 pyramidal cell and measured eIPSC by stimulating apical dendritic inhibition. We did not observe differences in the PPR (Figure 3D), 1/CV^2^ (Figure 3E), or intensity-amplitude curves recorded from the eIPSCs (Figure 3F). These findings further support the conclusion that social isolation neither affects the number of functional inhibitory synapses nor the presynaptic inhibitory neurotransmitter release mechanism at the perisomatic and apical dendritic site in both PH and non-PH cells.

### Social Isolation During the Juvenile Period Altered Excitability and Excitatory Synaptic Inputs of FS Interneurons

**Figure 4:**
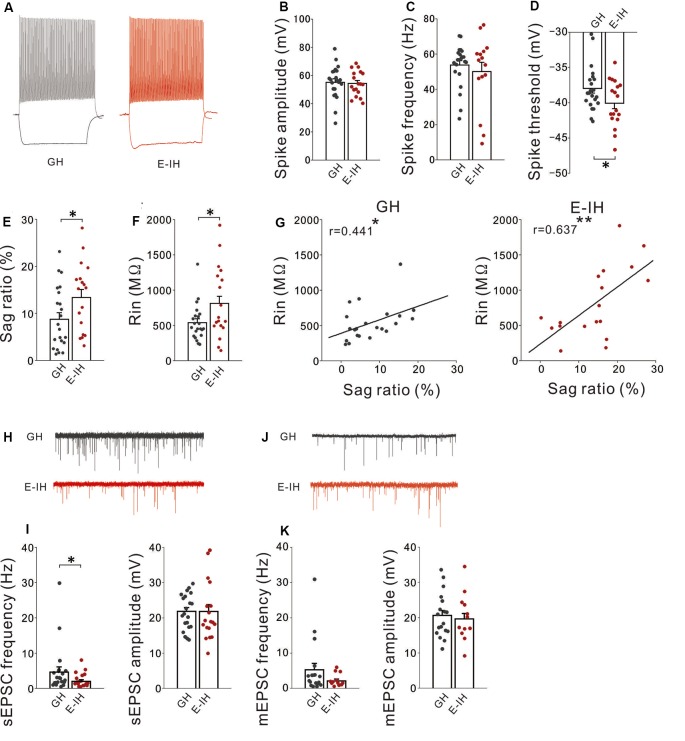
Social isolation increased the intrinsic excitability and decreased excitatory synaptic inputs onto fast-spiking (FS) interneurons. **(A)** Representative spikes of FS interneurons elicited by a current injection that was 100 pA larger than rheobase recorded in the group-housed (GH; left) and early isolation (E-IH) mice (right). **(B)** There was no significant between-group difference in the spike amplitude of FS interneurons (*t*_(38)_ = 0.234, *p* = 0.816; student’s *t*-test). **(C)** There was no significant between-group difference in the spike frequency (t_(38)_ = 0.637, *p* = 0.528; student’s *t*-test). **(D)** The spike threshold in the E-IH mice was significantly lower than that in the GH mice (*t*_(38)_ = 2.050, *p = 0.047; student’s *t*-test). **(E)** The sag ratio in the E-IH mice was significantly higher than that in the GH mice (*t*_(38)_ = −2.100, *p = 0.042; student’s *t*-test). **(F)** The input resistance in the E-IH mice was significantly higher than that in the GH mice (*t*_(38)_ = −2.215, *p = 0.033; student’s *t*-test). **(G)** There was a positive correlation between input resistance and sag ratio in the GH (left) and E-IH mice (right; GH: *r* = 0.441, *p < 0.05; E-IH: *r* = 0.637, **p < 0.01; Pearson correlation; number of cells in panels (**A–G**): 22 from 10 GH mice: 18 from nine E-IH mice). **(H)** Representative traces showing spontaneous excitatory postsynaptic currents (sEPSCs) recorded from FS cells in the GH and E-IH mice. **(I)** The sEPSC frequency in the E-IH mice was significantly lower than that in the GH mice (*U* = 126, *p = 0.046; Mann–Whitney *U* test). Moreover, there were no significant between-group differences in the sEPSC amplitude (*t*_(37)_ = 0.010, *p* = 0.992; student’s *t*-test; number of cells: 21 from 10 GH mice: 18 from nine E-IH mice). **(J)** Representative traces showing miniature EPSCs (mEPSCs) recorded from FS cells in the GH and E-IH mice. **(K)** There were no significant between-group differences in the mEPSC frequency (left) or amplitude (right; mEPSC frequency: *U* = 83, *p* = 0.305; Mann–Whitney *U* test, mEPSC amplitude: *t*_(37)_ = 0.284, *p* = 0.683; student’s *t*-test; number of cells: 18 from 10 GH mice: 12 from nine E-IH mice).

Given that there was no change in the inhibitory synapses and presynaptic inhibitory neurotransmitter release mechanism in PH cells, it is possible that increased sIPSC frequency in PH cells induced by social isolation might involve increased excitability of interneurons with inhibitory inputs onto PH cells. The neocortex hosts a diverse GABAergic interneuron population that collectively regulates the activity of principal neurons. Within this population, an interneuron subgroup population with an FS phenotype has powerful perisomatic-targeting inhibitory synapses on pyramidal cells, which frequently express the calcium-binding protein PV and present several distinct electrophysiological properties, including low input resistance, short membrane time constants, narrow action potentials, and a high maximum firing frequency in response to depolarizing current pulses (González-Burgos et al., 2005; Doischer et al., 2008). Therefore, we examined whether social isolation altered action potential properties in L5 FS interneurons in the mPFC. Prior to recording, we identified FS interneurons based on their action potential (“Materials and Methods” section Supplementary Figures S1, S4A). There were no significant between-group differences in the spike amplitude (Figure 4B) or frequency (Figure 4C). Notably, FS interneurons in the E-IH mice fired at a significantly lower spike threshold than those in the GH mice (Figure 4D). Previous studies have reported that PV+ cells (putative FS cells) expressed HCN4 (Hughes et al., 2013). Given the role of HCN channels in decreasing cellular excitability, it is possible that social isolation decreases the Ih current (expressed as sag-R) of FS cells, which results in a lowered spike threshold. Therefore, we investigated whether social isolation influenced the sag-R of L5 FS interneurons. We found a significantly larger sag-R and higher input resistance in FS interneurons in the E-IH mice (Figures 4E,F). Notably, there was a positive correlation between the sag-R and input resistance in both the GH and E-IH mice (Figure 4G), which was in contrast with the negative correlation observed between the sag-R and input resistance in L5 pyramidal cells (Supplementary Figures S2G, S3F). A larger sag-R indicates more h-current in FS interneurons, which decreases cellular excitability. It is possible that more excitable FS interneurons with lower spike thresholds and higher input resistance in the E-IH mice might paradoxically have a larger sag-R as a compensatory effect. To investigate whether these alterations simultaneously occur in FS interneurons, we evaluated the membrane properties of FS interneurons exposed to juvenile social isolation using 3-dimensional plots of these three values (Supplementary Figure S4A). Compared with the GH mice, we detected an FS cell subgroup in the E-IH mice that simultaneously had a low spike threshold, high input resistance, and large sag-R (Supplementary Figure S4A; right, circle). This suggests that juvenile social isolation increased the intrinsic excitability of FS interneurons, which led to a simultaneously lower spike threshold, increased input resistance, and increased sag-R in an FS interneuron subgroup in L5 of the mPFC.

FS interneuron activation mediated by excitatory inputs is crucial for pyramidal cell synchronization in the gamma frequency band related to cognitive function (Rotaru et al., 2011). Increased excitability of FS interneurons resulting from juvenile social isolation might be followed by increased excitatory synaptic inputs onto FS interneurons. Contrastingly, it might be followed by compensatory reduction of excitatory synaptic inputs onto FS interneurons. To assess these possibilities, we recorded sEPSCs on FS interneurons in both the GH and E-IH mice. We observed a significant decrease in the sEPSC frequency on FS interneurons in the E-IH mice (Figures 4H,I; left). Contrastingly, there were no significant differences in the sEPSC amplitudes in FS interneurons in the GH and E-IH mice (Figures 4H,I; right). Further, we analyzed the mEPSCs on FS interneurons and found that juvenile social isolation had no effect on the mEPSC frequency or amplitude in FS cells (Figures 4J,K). These findings suggest that juvenile social isolation decreased excitatory synaptic inputs onto FS cells. Similar to the aforementioned findings (Supplementary Figure S4A), we found that social isolation had a simultaneous effect on the spike threshold, sag-R, and sEPSC frequency in an FS interneuron subgroup (Supplementary Figure S4B; right). These findings suggest that social isolation also reduces the sEPSC frequency as a compensatory effect for increased FS interneuron excitability.

### Social Isolation During the Juvenile Period Did Not Alter Excitability and Excitatory Synaptic Inputs of Non-FS Interneurons

**Figure 5:**
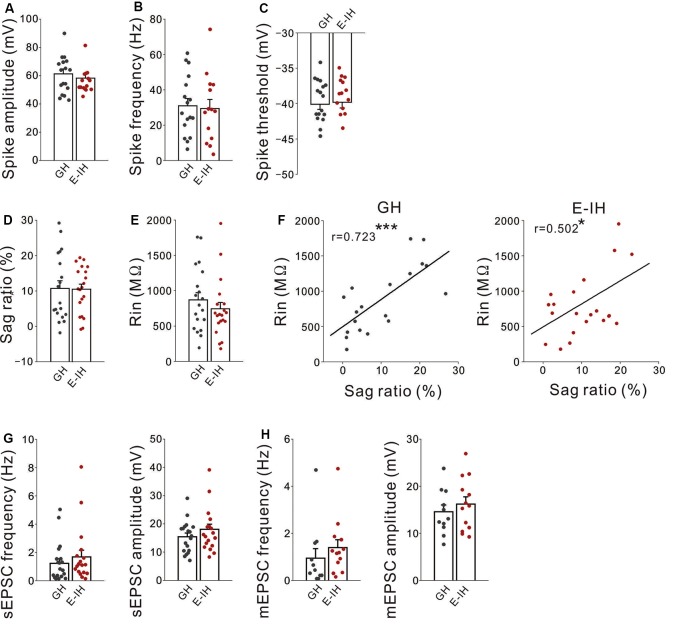
Social isolation did not alter excitatory inputs onto or excitability in non-fast spiking (non-FS) interneuron. **(A)** There was no significant between-group difference in the spike amplitude in non-FS interneurons (*t*_(29)_ = 1.252, *p* = 0.115; student’s *t*-test). **(B)** There was no significant between-group difference in the spike frequency (*t*_(29)_ = 0.240, *p* = 0.812; student’s *t*-test). **(C)** There was no significant between-group difference in the spike threshold (*t*_(29)_ = −0.524, *p* = 0.751; student’s *t*-test; number of cells in panels (**A–C**): 17 from 10 GH mice: 14 from nine E-IH mice). **(D)** There was no significant between-group difference in the sag ratio (*t*_(37)_ = 0.055, *p* = 0.957; student’s *t*-test). **(E)** There was no significant between-group difference in the input resistance (*t*_(37)_ = 0.880, *p* = 0.385; student’s *t*-test). **(F)** There was a positive correlation between the input resistance and sag ratio in the GH (left) and early isolation (E-IH) mice (right; GH; *r* = 0.723 ***p < 0.001, E-IH; *r* = 0.502 *p < 0.05; Pearson correlation; number of cells in panels **(D–F)**: 19 from 10 GH mice: 20 from nine E-IH mice). **(G)** There were no significant between-group differences in the spontaneous excitatory postsynaptic current (sEPSC) frequency (left) or amplitude (right; sEPSC frequency: *U* = 140; Mann–Whitney *U* test, *p* = 0.358, sEPSC amplitude: *t*_(35)_ = −1.165, *p* = 0.252; student’s *t*-test; number of cells: 19 from 10 GH mice: 18 from nine E-IH mice). **(H)** There were no significant between-group differences in the miniature EPSC (mEPSC) frequency (left) or amplitude (right; mEPSC frequency: *U* = 43; Mann–Whitney *U* test, *p* = 0.106, mEPSC amplitude: *t*_(22)_ = −0.762, *p* = 0.454; student’s *t*-test; number of cells: 11 from 10 GH mice: 13 from nine E-IH mice).

Next, to investigate whether juvenile social isolation had similar effects on L5 non-FS interneurons as those on L5 FS interneurons in the mPFC, the action potential properties of L5 non-FS interneurons in both the GH and E-IH mice was determined. There were no differences in the spike amplitude (Figure 5A), spike frequency (Figure 5B), spike threshold (Figure 5C), sag-R (Figure 5D), and input resistance (Figure 5E) between the GH and E-IH mice. However, similar to the FS interneurons, we found a positive correlation in the sag-R and input resistance in both the GH and E-IH mice (Figure 5F). There were no differences in the sEPSC frequency or amplitude of non-FS interneurons between the GH and E-IH mice (Figure 5G). Further, there were no differences in the mEPSC frequency and amplitude of non-FS interneurons between the GH and E-IH mice (Figure 5H). These findings suggest that social isolation does not affect the excitability of and excitatory synaptic inputs onto non-FS interneurons.

## Discussion

In this study, we demonstrated that 2 weeks of social isolation increased inhibitory synaptic activity and decreased intrinsic excitability in PH cells in the mPFC in the mice. We found that social isolation in the juvenile period between P21 and P35 was critical for the effects to be observed. The increase in the inhibitory synaptic inputs was neither due to alteration of neurotransmitter release mechanisms nor increased functional inhibitory synapses. Social isolation increased the intrinsic excitability of FS interneurons with lowered spike threshold and increased input resistance even though there was increased Ih and decreased excitatory synaptic inputs onto FS interneurons. Social isolation did not affect the excitability of non-FS interneurons. These results suggest that social isolation during the critical period enhances the activity of inhibitory neuronal circuits, including PH cells and FS interneurons in the mPFC in mice.

Sensory experience during the developmental period is considered important for the proper construction of neuronal circuits (Holtmaat and Svoboda, 2009). The observed enhanced inhibition of neuronal circuits by social isolation during development is consistent with previous findings regarding sensory deprivation during development (Maffei et al., 2006; Kannan et al., 2016). Visual deprivation during the critical period has been reported to enhance the amplitude of IPSCs mediated by FS interneurons (Maffei et al., 2006); further, within L4, GABAergic transmission strengthening is mediated by increased postsynaptic GABA receptor density and the density of readily releasable vesicles at interneuron terminals in the visual cortex (Nahmani and Turrigiano, 2014; Petrini et al., 2014). Moreover, GABAergic enhancement is mediated by an increase in both the number of postsynaptic GABAergic synapses and the probability of presynaptic GABA release (Kannan et al., 2016). Although sensory experience and social experience involve different information processing, lack of social experience during the juvenile period might disturb normal neuronal circuit development and induce excessive GABAergic inhibition through increased excitability of FS interneurons.

A previous study reported that juvenile social isolation reduced excitatory synaptic inputs onto PH cells but not onto non-PH cells (Yamamuro et al., 2018). In the present study, we found that juvenile social isolation increased inhibitory inputs onto PH cells but not non-PH cells. These findings indicate that juvenile social isolation shifts the excitation/inhibition (E/I) balance toward poor excitation and excessive inhibition in the synaptic inputs onto PH-cells. A tight balance between excitatory and inhibitory synaptic inputs onto neurons is critical for normal brain development and function. Contrastingly, imbalances between excitation and inhibition in synaptic transmission and neural circuits have been implicated in autism spectrum disorders (Cellot and Cherubini, 2014; Nelson and Valakh, 2015). The mPFC shares rich reciprocal connections with the mediodorsal (MD) thalamus (Condé et al., 1990; Ray and Price, 1992, 1993), which has been implicated in learning and memory, cognitive flexibility, and corollary discharge (Parnaudeau et al., 2015; Golden et al., 2016). Previous reports indicate that changes in thalamic volume (Tamura et al., 2010), as well as structural (Tan et al., 2010; Nair et al., 2013) and functional (Nair et al., 2013) connectivity between the PFC and MD thalamus can be observed in autism spectrum disorders. Social isolation induces autism-like behaviors in both humans and animals (Bicks et al., 2015). Several studies have reported that PH cells in the mPFC have axonal projections to subcortical regions including the thalamus (Dembrow et al., 2010; Gee et al., 2012; Lee et al., 2014). Moreover, previous studies have reported changes in the electrophysiological properties and synaptic connectivity of L5 neocortical pyramidal neurons in the autism model (Rinaldi et al., 2008a,b; Qiu et al., 2011; Kalmbach et al., 2015; Brumback et al., 2018; Yamamuro et al., 2018).Therefore, the observed disruption of the E/I balance in PH cells by social isolation in our study might indicate a potential mechanism for the decreased PFC-MD functional connectivity observed in human autism.

We found that social isolation increased inhibitory synaptic inputs onto PH cells, which was indicated by increased sIPSC frequency; however, it did not affect presynaptic neurotransmitter release mechanisms or the number of functional synapses, which we determined by analyzing the PPR, input-output curve, or 1/CV^2^ of the eIPSC amplitude on PH cells. These findings suggest that there could be an increase in the excitability of GABAergic interneurons as the source of inhibitory neurotransmission on PH cells. It is possible that FS cells are primary candidates for this GABAergic interneuron excitation by social isolation since it has been reported that FS PV interneurons preferentially inhibit PH cells compared with non-PH cells (Lee et al., 2014). Another possible explanation is that basket interneurons with FS features have somatic inhibition, which might have been preferentially detected since we performed whole-cell patch clamp recording from the soma of pyramidal cells (Freund and Katona, 2007). FS interneurons are primarily detected as PV+ cells. However, SST+ interneurons have been reported to have quasi FS patterns similar to those observed in PV+ interneurons (He et al., 2016). Therefore, we might have included SST interneuron with FS patterns as FS cells given the definition used in this study. Although, we did not detect alterations in the excitability and excitatory synaptic inputs of non-FS cells by social isolation, we cannot exclude the possibility that a subgroup of non-FS interneurons may have contributed to the increased inhibitory synaptic inputs induced by social isolation. Non-FS interneurons that were recorded could include subgroups of GABAergic interneurons that express cholecystokinin, SST, vasointestinal peptide, et cetera. For instance, one possible subgroup may be SST+ interneurons which are important for social exploration (Scheggia et al., 2020).

Previous studies have reported impaired E/I balance in the mPFC of autism-like mouse models with sociability deficits, which was similarly observed in the social isolation animal model (Makinodan et al., 2012). Correcting these imbalances has been reported to normalize key autistic-like phenotypes in these animals (Selimbeyoglu et al., 2017). Yang et al. (2018) revealed that Kv1.2 channel loss in basket cells exaggerated GABA release, which compromises firing activity in the cerebellum. Docosahexaenoic acid, an allosteric Kv1.2 agonist, has been reported to correct this dysregulated inhibition *in vitro*, as well as the acoustic startle reflex and social interaction *in vivo* in Fmr1-KO mice. Similarly, pharmacological inhibition of the excitability of FS interneurons might enhance the excitability of PH cells and improve mPFC function, and thus treat disorders induced by poor environments, such as social isolation.

## Data Availability Statement

All datasets generated for this study are included in the article/Supplementary Material.

## Ethics Statement

The animal study was reviewed and approved by The animal care and use committee of Nara Medical University.

## Author Contributions

HY and KY contributed to the conception and design of the study. KY, YO, HY, KO, and YN performed the experiments. KY analyzed the data. KY and HY wrote the article. MM, YS, and TK supervised the project. All authors contributed to manuscript revision, read, and approved the submitted version.

## Conflict of Interest

The authors declare that the research was conducted in the absence of any commercial or financial relationships that could be construed as a potential conflict of interest.
