# Supplementary material for: Juvenile Social Isolation Enhances the Activity of Inhibitory Neuronal Circuits in the Medial Prefrontal Cortex
Source: Front Cell Neurosci. 2020 May 12;14:105. doi: 10.3389/fncel.2020.00105 (PMC7235301; doi:10.3389/fncel.2020.00105)
Supplement: Supplementary file 1 [file Image_1.pdf]

Supplementary Material

1 Supplementary Figures

1.1 Supplementary Figure 1

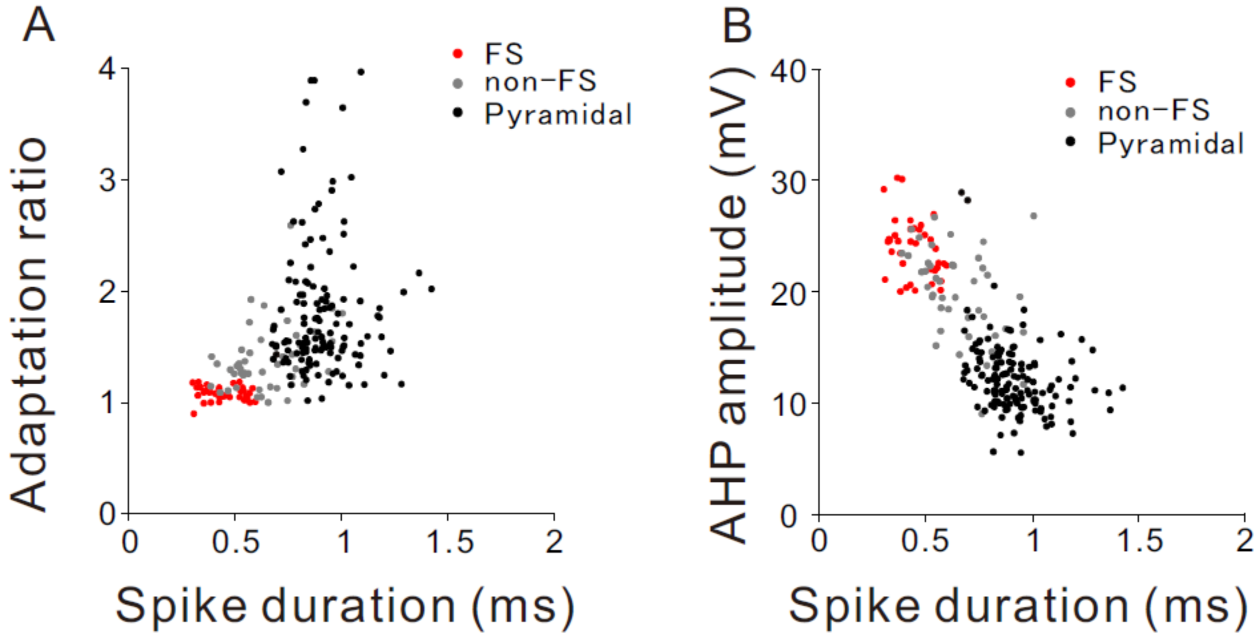

Supplementary Figure 1. We identified prefrontal cortex (PFC) pyramidal cells, fast-spiking (FS) interneurons, and non-fast spiking (non-FS) interneurons based on their firing patterns and morphological properties. **(A)** Left: the adaptation ratio (last interspike interval/first interspike interval, see Materials and Methods) plotted as a function of spike duration at half peak amplitude for 142 pyramidal cells (black), 38 FS neurons (red), and 45 non-FS cells (gray). Right: plot of the after hyperpolarization (AHP) amplitude versus spike duration at half-width. Cells classified as FS (see Materials and Methods) had a spike duration ≤ 0.6 ms, AHP amplitude ≥ 15 mV, and adaptation ratio ≤ 1.2.

1.2 Supplementary Figure 2

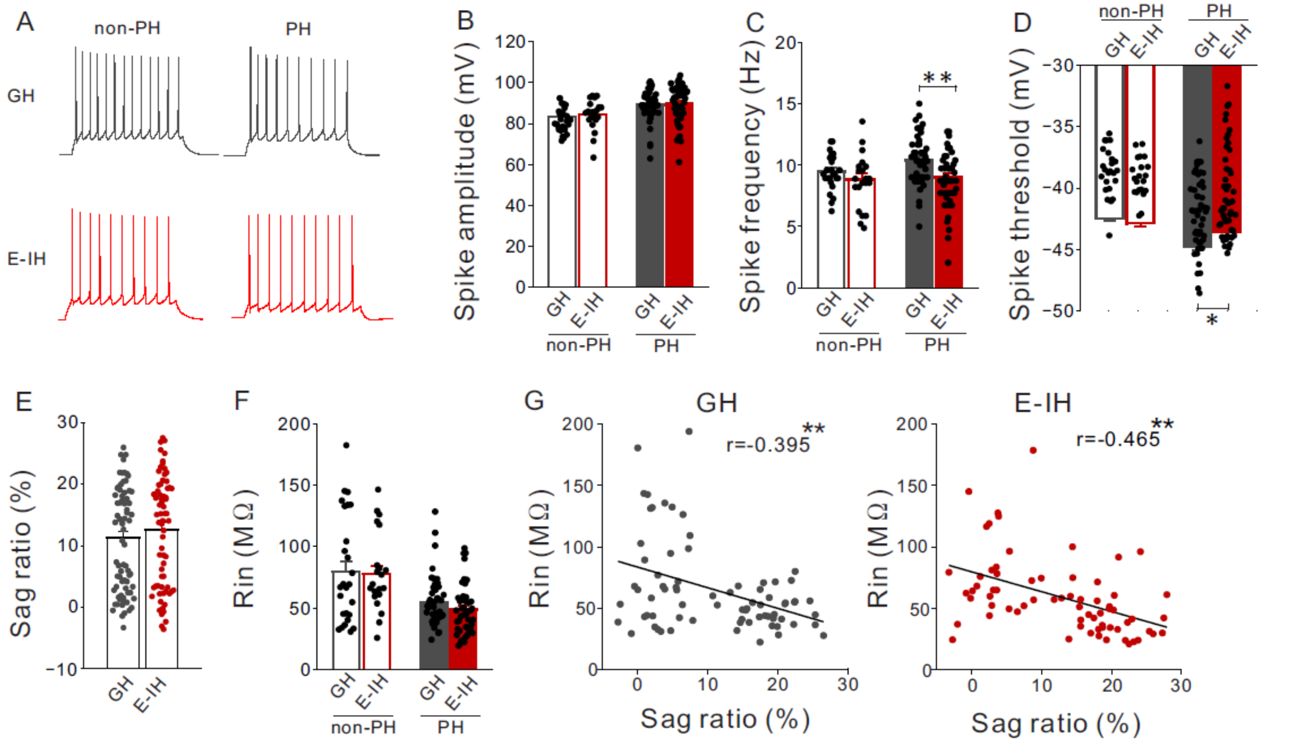

Supplementary Figure 2. Juvenile social isolation reduced intrinsic excitability only in prominent h-current (PH) cells but not non-PH cells. **(A)** Representative spike traces at 100pA injection recorded from PH (right) and non-PH cells (left) in the group-housing (GH) (upper) and early isolation housing (E-IH) (lower) mice. **(B)** There was no significant between-group difference in the spike amplitude in both PH and non-PH cells (2-way analysis of variance (ANOVA), effect of housing, $F_{(1,138)} = 0.417$, $p = 0.520$, effect of h-current, $F_{(1,138)} = 15.78$, $^{***}p < 0.001$, housing × h-current, $F_{(1,138)} = 0.0159$, $p = 0.901$). However, the spike amplitude in non-PH cells was significantly lower than that in PH cells. **(C)** For PH cells, the spike frequency in the E-IH mice was significantly lower than that in the GH mice (Tukey's HSD test: $p < 0.01$). However, for non-PH cells, there were no significant between-group differences in the spike frequency (2-way ANOVA, effect of housing, $F_{(1,138)} = 8$,$^{**}p = 0.005$, effect of h-current, $F_{(1,138)} = 2.246$, $p = 0.136$, housing × h-current, $F_{(1,138)} = 1.1$, $p = 0.296$). **(D)** For PH cells, the spike threshold in the E-IH mice was significantly higher than that in the GH mice (Tukey's HSD test: $P < 0.05$). However, for non-PH cells, there were no significant between-group differences in the spike threshold. However, the spike threshold in non-PH cells was significantly higher than that in PH cells in the GH mice (2-way ANOVA, effect of housing, $F_{(1,138)} = 1.167$, $p = 0.282$, effect of h-current, $F_{(1,138)} = 18.71$, $^{***}p < 0.001$, housing × h-current, $F_{(1,138)} = 5.452$, $^{*}p = 0.02$). (Number of cells in B, C, D: 25 non-PH and 48 PH from 6 GH mice: 21 non-PH and 48 PH from 6 E-IH mice) **(E)** There was no significant between-group

difference in the sag ratio of layer (L) 5 pyramidal cells (t_{141} = -1.025, p = 0.767; Student's t-test). (Number of cells: 73 from 6 GH mice / 69 from 6 E-IH mice) (**F**) There was no significant between-group difference in the input resistance in either PH or non-PH cells. However, the input resistance in non-PH cells was significantly higher than that in PH cells (2-way ANOVA, effect of housing, $F_{(1,138)} = 0.498$, p = 0.481, effect of h-current, $F_{(1,138)} = 29.39$, ***p < 0.001, housing × h-current, $F_{(1,138)} = 0.117$, p = 0.733). (Number of cells: 25 non-PH and 48 PH from 6 GH mice: 21 non-PH and 48 PH from 6 E-IH mice) **(G)** There was a significant negative correlation between input resistance and sag ratio in both the GH mice (left) and the E-IH mice (right) (GH; r = -0.395 **p < 0.01, E-IH; r = -0.465 **p < 0.01; Pearson correlation). (Number of cells: 73 from 6 GH mice: 69 from 6 E-IH mice)

1.3 Supplementary Figure 3

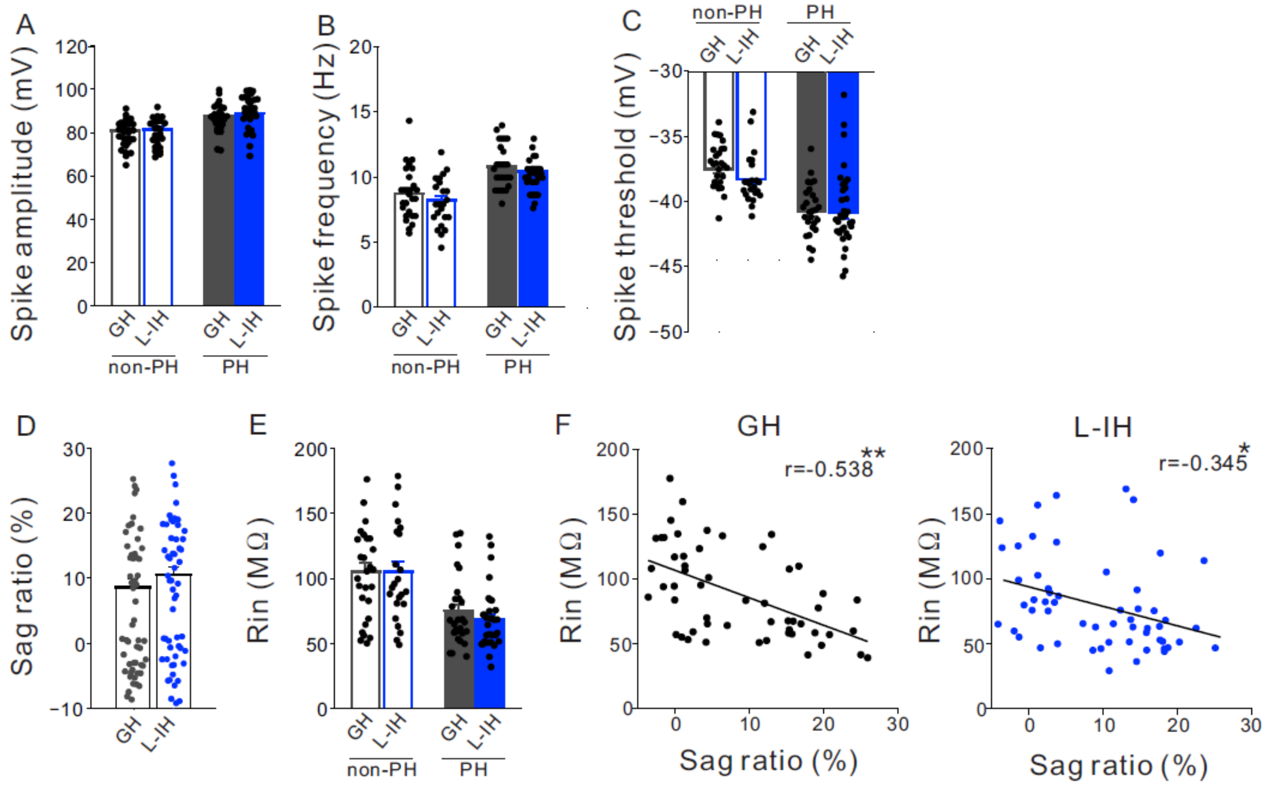

Supplementary Figure 3. Late social isolation did not alter intrinsic excitability of layer (L) 5 pyramidal cell. **(A)** There was no significant between-group difference in the spike amplitude in prominent h-current (PH) or non-PH cells. However, the spike amplitude in non-PH cells was significantly lower than that in PH cells (2-way analysis of variance (ANOVA), effect of housing, $F_{(1,106)} = 0.732$, p = 0.394, effect of h-current, $F_{(1,106)} = 30.05$, ***p < 0.001, housing × h-current, $F_{(1,106)} = 0.070$, p = 0.792). **(B)** There was no significant between-group difference in the spike frequency in PH or non-PH cells. However, the spike number in non-PH cells was significantly lower than that in PH cells (2-way ANOVA, effect of housing, $F_{(1,106)} = 2.288$, p = 0.133, effect of h-current, $F_{(1,106)} = 46.95$, ***p < 0.001, housing × h-current, $F_{(1,106)} = 0.059$, p = 0.808). **(C)** There was

no significant between-group difference in the spike threshold in the PH or non-PH cells. However, the spike threshold in non-PH cells was significantly higher than that in PH cells (2-way ANOVA, effect of housing, $F_{(1,106)} = 0.873$, $p = 0.352$, effect of h-current, $F_{(1,106)} = 44.92$, ***$p < 0.001$, housing × h-current, $F_{(1,106)} = 0.781$, $p = 0.379$). (Number of cells in A, B, C: 27 non-PH and 27 PH from 6 GH mice: 23 non-PH and 33 PH from 6 L-IH mice) **(D)** There was no significant between-group difference in the sag ratio of L5 pyramidal cells ($t_{108} = -1.159$, $p = 0.249$; Student's t-test). (Number of cells: 54 from 6 GH mice: 56 from 6 L-IH mice) **(E)** There was no significant between-group difference in the input resistance in PH or non-PH cells. However, the input resistance in non-PH cells was significantly higher than that in PH cells (2-way ANOVA, effect of housing, $F_{(1,106)} = 0.297$, $p = 0.587$, effect of h-current, $F_{(1,106)} = 31.74$, ***$p < 0.001$, housing × h-current, $F_{(1,106)} = 0.303$, $p = 0.583$). (Number of cells: 27 non-PH and 27 PH from 6 GH mice: 23 non-PH and 33 PH from 6 L-IH mice) (F) There was a significant negative correlation between input resistance and sag ratio in both the group-housing (GH) mice (left) and early isolation (L-IH) mice (right) (GH; $r = -0.538$ **$p < 0.01$, L-IH; $r = -0.345$ *$p < 0.05$; Pearson correlation). (Number of cells: 54 from 6 GH mice : 56 from 6 L-IH mice)

1.4 Supplementary Figure 4

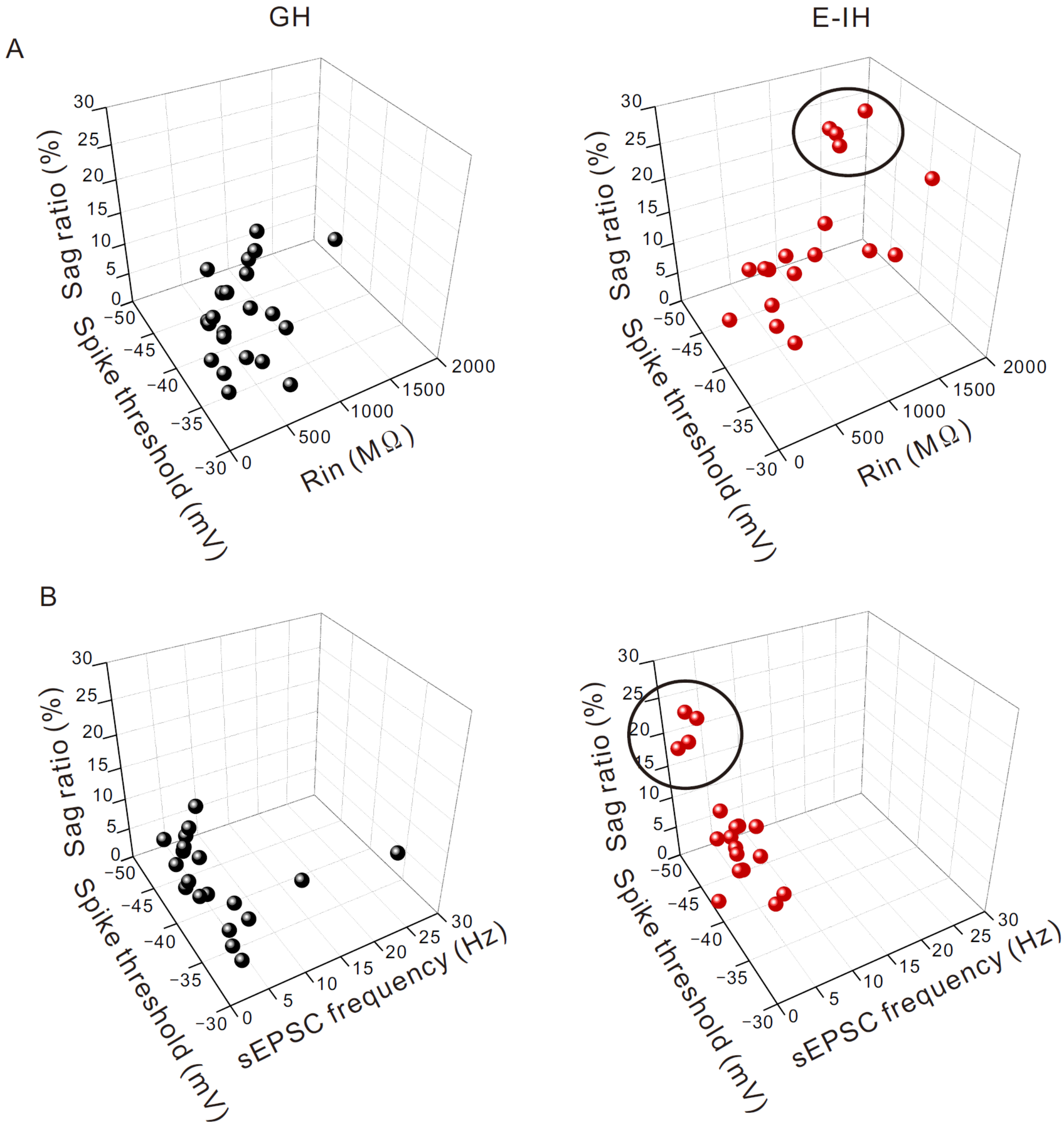

Supplementary Figure 4. The social isolation-induced alterations of factors relative to intrinsic excitability simultaneously occur in one fast-spiking (FS) interneuron. **(A)** Each FS interneuron plotted according to their values of spike threshold, sag ratio, and input resistance in a three-dimensional graph. In the early isolation (E-IH) mice (right), there was an FS interneuron subgroup (shown in a circle) that simultaneously had lower spike threshold, larger sag ratio, and higher input resistance, compared with those in the group-housing (GH) mice. **(B)** Each FS interneuron plotted according to their values of spike threshold, sag ratio, and spontaneous excitatory postsynaptic

current (sEPSC) frequency in a three-dimensional graph. In the E-IH mice (right), there is an FS interneuron subgroup (shown in a circle) that simultaneously had lower spike threshold, larger sag ratio, and lower sEPSC frequency, compared with those in the GH mice.
